# Supplementary material for: Programmable Single and Multiplex Base-Editing in Bombyx mori Using RNA-Guided Cytidine Deaminases
Source: G3 (Bethesda). 2018 Mar 19;8(5):1701–9. doi: 10.1534/g3.118.200134 (PMC5940161; doi:10.1534/g3.118.200134)
Supplement: Supplementary file 1 [file 1701FileS1.pdf]

Figure S1.

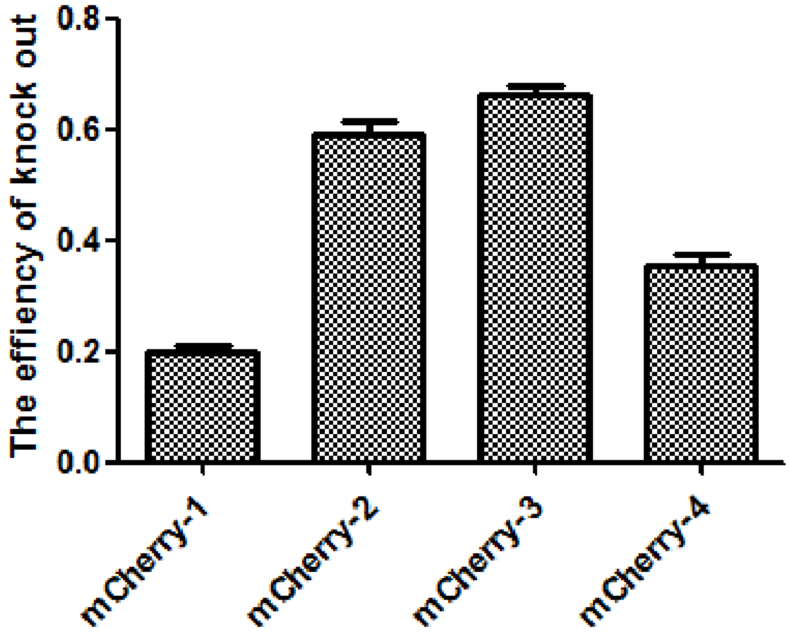

Figure S1. The efficiencies of *mCherry* being knocked out.

Efficiency for each gRNA is shown by comparing the percent of mCherry-negative cells with control. The heights of bar graphs show the mean of three independent replicates.

Table S1. Design of gRNA sequences

gRNAs	sequence
Blos2	GGAACACTACATGCTGCTTGAGG
Yellow-e	GAGTCGCACTCACCGTAGCTAGG
mCherry-1	GCACCCAGACCGCCAAGCTGAAGG
mCherry-2	GTGTCCCAGGCGAAGGGCAGGGGG
mCherry-3	GCTCCCACTTGAAGCCCTCGGGG
mCherry-4	GACCCAGGACTCCTCTCTGCAGG
Puromycin-1	GCCGCTCAACAGCAAATGGAAGG
Puromycin-2	GAGTTGTCCAGACAGCAACAGCGG
GAPDH-1	GCTACACAGAAAACTGTTGATGG
GAPDH-2	GTCACGCCATAATTTTCCAGAAGG
V-ATPase B-1	GAGGGTCAGCCCATCAACCCATGG
V-ATPase B-2	GTACAGCTAAAGCTTGCAGATGG
EGFP-gRNA1	GAGGGCGAGGAGCTGTTCACCGGG
EGFP-gRNA2	GCGAGGAGCTGTTCACCGGGGTGG
EGFP-gRNA3	GGTGCCCATCCTGGTCGAGCTGG
EGFP-gRNA4	GCCCATCCTGGTCGAGCTGGACGG
EGFP-gRNA5	GCCGTCCAGCTCGACCAGGATGG
EGFP-gRNA6	GCGTCGCCGTCCAGCTCGACCAGG
EGFP-gRNA7	GCAAGTTCAGCGTGTCCGGCGAGG
EGFP-gRNA8	GCAGCGTGTCCGGCGAGGGCGAGG

EGFP-gRNA9	GCAGGGTCAGCTTGCCGTAGGTGG
EGFP-gRNA10	GCAGGGTCAGCTTGCCGTAGGTGG
EGFP-gRNA11	GCCAGGGCACGGGCAGCTTGCCGG
EGFP-gRNA12	GTGGTCACGAGGGTGGGCCAGGG
EGFP-gRNA13	GGTCAGGGTGGTCACGAGGGTGG
EGFP-gRNA14	GTAGGTCAGGGTGGTCACGAGGG
EGFP-gRNA15	GCGTAGGTCAGGGTGGTCACGAGG
EGFP-gRNA16	GCTGCACGCCGTAGGTCAGGGTGG
EGFP-gRNA17	GCACTGCACGCCGTAGGTCAGGG
EGFP-gRNA18	GCTGAAGCACTGCACGCCGTAGG
EGFP-gRNA19	GCTTCATGTGGTCGGGGTAGCGG
EGFP-gRNA20	GCGTGCTGCTTCATGTGGTCGGGG
EGFP-gRNA21	GAGAAGTCGTGCTGCTTCATGTGG
EGFP-gRNA22	GCATGCCCGAAGGCTACGTCCAGG
EGFP-gRNA23	GCTGGACGTAGCCTTCGGGCATGG
EGFP-gRNA24	GACCATCTTCTTCAAGGACGACGG
EGFP-gRNA25	GCCGTCGTCCTTGAAGAAGATGG
EGFP-gRNA26	GCAACTACAAGACCCGCGCCGAGG
EGFP-gRNA27	GCTCGAACTTCACCTCGGCGCGGG
EGFP-gRNA28	GTCGCCCTCGAACTTCACCTCGG
EGFP-gRNA29	GAAGTTCGAGGGCGACACCCTGG
EGFP-gRNA30	GCAGCTCGATGCGGTTCACCAGGG

EGFP-gRNA31	GTCAGCTCGATGCGGTTCACCAGG
EGFP-gRNA32	GCGATGCCCTTCAGCTCGATGCGG

All the gRNA sequences have been used in this experiment were displayed in the table above. The letters with underline represent PAM sequence.

Table S2. Primers for PCR amplification in targeted genomic regions

primer	Sequence (5’-3’)
Blos2-PCR-F	TCCAATTTGAGGGCAATGCTAC
Blos2-PCR-R	ATTTCACCACCTCATTCAACTAAGAT
Yellow-e-PCR-F	GTACAATTTGTCCCAACCTATCT
Yellow-e-PCR-R	GCCCTTGACCTTTACTATTATGT
mCherry-PCR-F	AACTCCGCCATCATCAAGGA
mCherry-PCR-R	TGGTGTAGTCCTCGTTGTGGG
Puromycin-PCR-F	GACATTGGAAAGGTCTGGGTG
Puromycin-PCR-R	CAGTCTTTCGTAGAACGGGAGG
GAPDH-PCR-F	GTCGCTATAAATGACCCTTTCAT
GAPDH-PCR-R	AGCTGCCTCCTTGACCTTTT
V-ATPase B-1-PCR-F	GTCACTAAGGACTGCCGAAGA
V-ATPase B-1-PCR-R	AGCACAAGAGTGATACGAGCC
V-ATPase B-2-PCR-F	AAATTACAGGCAGAGCAAGT
V-ATPase B-2-PCR-R	CATATGCACCATAATTGACCTAC
EGFP-PCR-1F	TAAATACAGCCCGCAACGAT
EGFP-PCR-1R	GTCAGCTTGCCGTAGGTGG
EGFP-PCR-2F	ATGGTGAGCAAGGGCGAG
EGFP-PCR-2R	GTCAGGGTGGTCACGAGGG
EGFP-PCR-3F	AAACGGCCACAAGTTCAGCG
EGFP-PCR-3R	AAGAAGTCGTGCTGCTTCAT

EGFP-PCR-4F	CAAGCTGCCCGTGCCCTG
EGFP-PCR-4R	GCGGGTCTTGTAGTTGC
EGFP-PCR-5F	CAGTGCTTCAGCCGCTACCC
EGFP-PCR-5R	GGATGTTGCCGTCCTCCTTG
EGFP-PCR-6F	ATGGTGAGCAAGGGCGAG
EGFP-PCR-6R	CTCGCCCTTGCTCACCAT

In the list, all the primers have been used in this experiment were displayed in the table above.

Table S3. Indel frequencies for 32 gRNAs target *EGFP*

gRNA	1	2	3	4	5	6	7	8	9	10	11	12	13	14	15	16	control
Frequency of indel (%)	2.5	3.1	2.7	2.4	2.2	2.6	3.4	4.0	8.1	2.8	1.5	1.9	1.0	1.0	1.1	1.1	
gRNA	17	18	19	20	21	22	23	24	25	26	27	28	29	30	31	32	1.2
Frequency of indel (%)	1.1	1.0	1.6	1.4	0.5	5.0	1.2	2.5	1.3	2.0	1.9	1.9	1.9	3.7	1.5	2.4	

gRNA	32 gRNAs all together	control
Frequency of indel (%)	0.6	0.6

The upper table shows the indel frequency for every gRNA that transfected with BE3 one by one. The under table shows the indel frequency for the whole of 32 gRNAs that transfected with BE3.
